# Vitamin-D-Supplementierung

**DOI:** 10.1007/s00108-022-01435-4

**Published:** 2022-12-08

**Authors:** Heike A. Bischoff-Ferrari, Sirka Nitschmann

**Affiliations:** 1 Universitäre Altersmedizin und Altersforschung, Universitätsspital Zürich, Universität Zürich, Stadtspital Zürich, Zürich, Schweiz; 2 Lippetal, Deutschland

## Originalliteratur

LeBoff MS, Chou AH, Ratliff KA et al (2022) Supplemental vitamin D and incident fractures in midlife and older adults. N Engl J Med 387:299-309

Frakturen sind ein großes öffentliches Gesundheitsproblem. In den USA leiden etwa 53,6 Mio. Menschen unter Osteoporose und/oder geringer Knochenmasse und sind somit vermehrt frakturgefährdet. Vitamin-D-Präparate werden häufig als Nahrungsergänzung für die Knochengesundheit empfohlen. Obwohl die Daten zum Sinn einer Supplementierung uneinheitlich sind, hat sich die Einnahme von Vitamin-D-Präparaten von 1999 bis 2012 in den USA von 5,1 % auf 19 % erhöht.

Ziel der im Folgenden vorgestellten Studie war es, den Stellenwert der Vitamin-D-Supplementierung bei allgemein gesunden Menschen im mittleren bis höheren Lebensalter zu verifizieren.
