# Supplementary material for: Vitamin D supplementation
Source: Inn Med (Heidelb). 2022 Dec 8;64(1):107–10. [Article in German] doi: 10.1007/s00108-022-01435-4 (PMC9852139; doi:10.1007/s00108-022-01435-4)
Supplement: Related texts

# Research article: Zusammenfassung der Studie

**Authors:** Sirka Nitschmann

**Affiliations:** Lippetal, Deutschland

## Studiendesign

Retrospektive Zusatzauswertung von Patienten, die während einer randomisierten, doppelblinden, kontrollierten, 2 × 2-faktoriellen^1^ Studie (VITAL-Studie^2^, Follow-up-Zeit 5,3 Jahre) eine Fraktur erlitten haben.

### Einschlusskriterien

- Teilnehmer der VITAL-Studie, die Frakturen erlitten haben

### Endpunkte

#### Primär.

- Frakturen insgesamt sowie nichtvertebrale und Hüftfrakturen

#### Sekundär.

- Frakturen insgesamt sowie nichtvertebrale und Hüftfrakturen ohne Zehen‑/Finger‑/Schädel-, periprothetische und pathologische Frakturen
- Bestätigte Frakturen mit Ausnahme von periprothetischen und pathologischen Frakturen:
  - Schwere osteoporotische Frakturen
  - Beckenfraktur
  - Handgelenkfraktur

## Methodik

Die Teilnehmer der VITAL-Studie füllten jährlich einen Fragebogen aus, in dem Frakturen gemeldet werden sollten. Patienten, die eine Fraktur erlitten hatten, erhielten einen weiteren Fragebogen, in dem Informationen zur Fraktur sowie ein möglicher Zusammenhang mit vorausgegangener Prothesenversorgung und Malignomen abgefragt wurden und die Erlaubnis zur Einsichtnahme in die Krankenakten erteilt wurde. Alle Frakturen wurden zentral von Studienärzten bzw. -mitarbeitern beurteilt, die nicht wussten, welcher VITAL-Studiengruppe die Patienten angehörten. Die Röntgenbilder von Hüft- und Oberschenkelfrakturen wurden von einem Radiologen anhand von Kriterien der American Society for Bone and Mineral Research beurteilt, um periprothetische und atypische Frakturen ausschließen zu können.

## Ergebnisse

Von den 25.871 Teilnehmern der VITAL-Studie – knapp über die Hälfte waren Frauen und das Durchschnittsalter betrug 67,1 Jahre – erlitten 1551 Patienten während eines mittleren Follow-up-Zeitraums von 5,3 Jahren 1991 Frakturen.

Hinsichtlich des primären Endpunkts konnte kein signifikanter Vorteil für die Vitamin-D-Gabe gezeigt werden (Tab. 1).

**Table 1:** 

	Vitamin-D-Patienten (*n* = 12.927)	Placebopatienten (*N* = 12.944)	Hazard Ratio (95 %-Konfidenzintervall)	*p*-Wert
**Primärer Endpunkt**				
Gesamtfrakturzahl	769	782	0,98 (0,89–1,08)	*p* = 0,70
Nichtvertebrale Frakturen	721	744	0,97 (0,87–1,07)	*p* = 0,50
Hüftfrakturen	57	56	1,01 (0,70–1,47)	*p* = 0,96
**Sekundäre Endpunkte**				
Gesamtfrakturzahl	678	685	0,99 (0,89–1,10)	–
Nichtvertebrale Frakturen	630	649	0,97 (0,87–1,08)	–
Hüftfrakturen	54	52	1,03 (0,70–1,52)	–
Bestätigte Frakturen mit Ausnahme von periprothetischen und pathologischen Frakturen	–	–	–	–
– Schwere osteoporotische Frakturen	276	278	0,99 (0,83–1,17)	–
– Beckenfraktur	32	29	1,08 (0,64–1,80)	–
– Handgelenkfraktur	118	132	0,89 (0,69–1,15)	–

Die Variablen „Alter“, „Geschlecht“, „Ethnische Gruppe“, „Body-Mass-Index“ oder „25-Hydroxyvitamin-D-Serumausgangsspiegel“ hatten keinen Einfluss auf das Ergebnis, ebenso wenig zusätzlich seitens der Patienten eingenommene Kalzium- oder Vitamin-D-Präparate. Es konnten keine signifikanten Auswirkungen auf die Frakturhäufigkeit durch 25-Hydroxyvitamin-D-Grenzwerte (< 12, < 20, < 30 oder ≥ 50 ng/ml), den Serumkalziumspiegel und den Parathormonspiegel gezeigt werden, auch vorausgegangene Fragilitätsfrakturen und/oder die Einnahme von Osteoporosetherapeutika hatten keinen Einfluss auf die Frakturhäufigkeit.

Bei den unerwünschten Ereignissen, wie Hyperkalzämie oder Nierensteinen, die in der Hauptstudie untersucht wurden, gab es keine wesentlichen Unterschiede zwischen den Gruppen.

### Take home message

In der VITAL-Studie senkte eine Vitamin-D3-Supplementierung mit 2000 IE/Tag das Frakturrisiko bei allgemein gesunden Erwachsenen im mittleren und höheren Lebensalter, die keinen Vitamin-D-Mangel und keine geringe Knochenmasse bzw. Osteoporose aufwiesen, nicht signifikant. Aber es wäre kurzsichtig, aufgrund dieser Daten älteren Erwachsenen mit einem hohen Risiko für einen Vitamin-D-Mangel die in den heutigen Leitlinien empfohlene Dosis von 600 bis 800 IE/Tag Vitamin D vorzuenthalten.

# Research article: Kommentar

**Authors:** Heike A. Bischoff-Ferrari

**Affiliations:** grid.7400.3 0000 0004 1937 0650 Universitäre Altersmedizin und Altersforschung, Universitätsspital Zürich, Universität Zürich, Stadtspital Zürich, Zürich, Schweiz

Dr. Meryl S. LeBoff, Erstautorin des Manuskripts zu den Frakturdaten der VITAL-Studie, fasst die Ergebnisse sehr gut zusammen [1]: Eine Vitamin-D3-Supplementierung mit 2000 IE/Tag führt bei allgemein gesunden Erwachsenen im mittleren und höheren Lebensalter, die keinen Vitamin-D-Mangel haben und nicht bezüglich geringer Knochenmasse oder Osteoporose ausgewählt wurden, nicht zu einem signifikant niedrigeren Frakturrisiko als ein Placebo.

## Stellt das Resultat der VITAL-Studie damit die aktuellen Leitlinien zu Vitamin D infrage?

Nein, weil die Dosierung von 600 bis 800 IE/Tag in den aktuellen Leitlinien auf die Behebung eines Vitamin-D-Mangels abzielt sowie bei vulnerablen älteren Erwachsenen mit Vitamin-D-Mangel deren Fraktur- und Sturzrisiko vermindert. Letztere Empfehlung basiert unter anderem auf Daten von über 30.000 älteren Erwachsenen mit Vitamin-D-Mangel, die in randomisierten Doppelblindstudien mit einer überwiegend täglichen Gabe von 800 IE Vitamin D untersucht wurden und eine 30 %ige Verminderung von Hüftfrakturen aufwiesen [2]. Zudem wurde 2011 in der Leitlinie des Institute of Medicine (IOM) eine Vitamin-D-Tagesdosis von 600 bis 800 IE empfohlen – als diejenige Zufuhr, die den Vitamin-D-Mangel und den Bedarf für die Knochengesundheit bei 97,5 % der Bevölkerung zu decken vermag [3]. Diese Empfehlung wurde als volksgesundheitlich präventive Zufuhr definiert, die keine Blutspiegelmessung von 25-Hydroxyvitamin D vor Start einer Vitamin-D-Supplementierung voraussetzt [3].

Die untersuchte Dosis von Vitamin D in VITAL betrug 2000 IE/Tag, und die Untersuchungsgruppe bestand aus gesunden mittelalten und älteren Erwachsenen ohne Vitamin-D-Mangel. VITAL war als Primärpräventionsstudie geplant und schloss generell gesunde Menschen im Alter von 50 Jahren und darüber ein. Zu Beginn der Studie hatten nur 12,7 % einen Vitamin-D-Mangel mit 25-Hydroxyvitamin-D-Blutwerten < 20 ng/ml [4] und 42,6 % der Studienteilnehmer nahmen zu Studienbeginn bereits 800 IE Vitamin D ein [1]. Dazu erlaubte VITAL, die Eigensupplementierung von Vitamin D zusätzlich zur Studienmedikation im Umfang von 800 IE/Tag während der Studie weiterzuführen [4].

Die Autoren stellen diese Rahmenbedingungen und Limitationen in ihrer Diskussion korrekt dar, allerdings führte das begleitende Editorial wegen seiner auffallend engen Sichtweise zu Verunsicherung bei Ärzten in Klinik und Praxis [5]. Die pauschale Aussage „Vitaminpräparate haben in der Allgemeinbevölkerung älterer Erwachsener keinen bedeutenden gesundheitlichen Nutzen“ als Zusammenfassung der Frakturdaten aus VITAL ist zu kurzsichtig. Die Autoren des Editorials ignorieren damit die Tatsache, dass immer noch ein Großteil der Weltbevölkerung und insbesondere ältere Erwachsene eine unzureichende Vitamin-D-Versorgung haben. Die Behauptung, anhand von VITAL ausschließen zu können, dass Vitamin D auch bei Menschen mit Vitamin-D-Mangel einen Vorteil bezüglich des Frakturrisikos bringe, ist angesichts der kleinen Subgruppe an Patienten mit Vitamin-D-Mangel in VITAL und entsprechend weniger Frakturen nicht haltbar.

Eine falsche Interpretation der VITAL-Daten könnte Schaden anrichten, indem sie Ärzte oder ältere Erwachsene selbst dazu ermutigt, die Vitamin-D-Supplemente abzusetzen. Wir würden damit einen belegten und kostengünstigen volksgesundheitlichen Ansatz zur Senkung der weltweiten Osteoporosebelastung aufgeben.

Daneben haben neuere Metaanalysen mit Einschluss von intermittierenden hoch dosierten Bolusgaben ebenfalls zur aktuellen Verunsicherung bezüglich der Vitamin-D-Supplementierung beigetragen [6]. Tatsächlich hissen diese oft mit hoher Teilnehmerzahl durchgeführten Studien seit 2007 eine rote Flagge in der Forschungsliteratur [6]. Smith et al. zeigten bereits 2007 eine 49 %ige Zunahme des Hüftbruchrisikos bei jährlich 300.000 IE Vitamin D [7], dann zeigten Sanders et al. 2010 eine 16 %ige Zunahme des Sturzrisikos und eine 26 % Zunahme des Frakturrisikos bei jährlich 500.000 IE Vitamin D [8], Ginde [9] und Khaw [10] zeigten 2017, dass das Risiko von Stürzen und Frakturen bereits bei 100.000 IE Vitamin D im Monat ansteigt oder kein Nutzen erzielt wird. Ein ähnliches Muster zeigte sich für die monatliche Bolusgabe von 60.000 IE mit einer Zunahme des Sturzrisikos bei vulnerablen (2016 [11]) und ohne Nutzen bei nichtselektionierten älteren Erwachsenen in D‑HEALTH (2022 [12]).

Zusammengefasst erscheint es zu kurzsichtig, anhand der VITAL-Studie oder der Studien mit Vitamin-D-Bolusgabe älteren Erwachsenen mit einem hohen Risiko für einen Vitamin-D-Mangel die in den heutigen Leitlinien empfohlene tägliche Dosis von 600 bis 800 IE Vitamin D am Tag vorzuenthalten.
